# Novel Approach for Evaluation of *Bacteroides fragilis* Protective Role against *Bartonella henselae* Liver Damage in Immunocompromised Murine Model

**DOI:** 10.3389/fmicb.2016.01750

**Published:** 2016-11-07

**Authors:** Chiara Pagliuca, Annunziata G. Cicatiello, Roberta Colicchio, Adelaide Greco, Raimondo Cerciello, Luigi Auletta, Sandra Albanese, Elena Scaglione, Caterina Pagliarulo, Gabiria Pastore, Gelsomina Mansueto, Arturo Brunetti, Bice Avallone, Paola Salvatore

**Affiliations:** ^1^Department of Molecular Medicine and Medical Biotechnology, Federico II University Medical SchoolNaples, Italy; ^2^CEINGE-Advanced BiotechnologiesNaples, Italy; ^3^Department of Advanced Biomedical Science, Federico II University Medical SchoolNaples, Italy; ^4^Institute of Biostructure and Bioimaging, National Research CouncilNaples, Italy; ^5^Department of Biology, University of Naples Federico IINaples, Italy; ^6^IRCCS SDNNaples, Italy; ^7^Department of Sciences and Technologies, University of SannioBenevento, Italy

**Keywords:** *Bartonella henselae*, *Bacteroides fragilis*, PSA, SCID mice, HFUS, Histological examination, Imaging, TEM

## Abstract

*Bartonella henselae* is a gram-negative facultative intracellular bacterium and is the causative agent of cat-scratch disease. Our previous data have established that *Bacteroides fragilis* colonization is able to prevent *B. henselae* damages through the polysaccharide A (PSA) in an experimental murine model. In order to determine whether the PSA is essential for the protection against pathogenic effects of *B. henselae* in immunocompromised hosts, SCID mice were co-infected with *B. fragilis* wild type or its mutant *B. fragilis* ΔPSA and the effects of infection on murine tissues have been observed by High-Frequency Ultrasound (HFUS), histopathological examination, and Transmission Electron Microscopy (TEM). For the first time, echostructure, hepatic lobes length, vascular alterations, and indirect signs of hepatic dysfunctions, routinely used as signs of disease in humans, have been analyzed in an immunocompromised murine model. Our findings showed echostructural alterations in all infected mice compared with the Phosphate Buffer Solution (PBS) control group; further, those infected with *B. henselae* and co-infected with *B. henselae/B. fragilis* ΔPSA presented the major echostructural alterations. Half of the mice infected with *B. henselae* and all those co-infected with *B. henselae/B. fragilis* ΔPSA have showed an altered hepatic echogenicity compared with the renal cortex. The echogenicity score of co-infected mice with *B. henselae/B. fragilis* ΔPSA differed significantly compared with the PBS control group (p < 0.05). Moreover the inflammation score of the histopathological evaluation was fairly concordant with ultrasound findings. Ultrastructural analysis performed by TEM revealed no significant alterations in liver samples of SCID mice infected with *B. fragilis* wild type while those infected with *B. fragilis* ΔPSA showed the presence of collagen around the main vessels compared with the PBS control group. The liver samples of mice infected with *B. henselae* showed macro-areas rich in collagen, stellate cells, and histiocytic cells. Interestingly, our data demonstrated that immunocompromised SCID mice infected with *B. henselae* and co-infected with *B. henselae/B. fragilis* ΔPSA showed the most severe morpho-structural liver damage. In addition, these results suggests that the HFUS together with histopathological evaluation could be considered good imaging approach to evaluate hepatic alterations.

## Introduction

*Bartonella henselae*, a facultative intracellular gram-negative bacterium, causes cat-scratch disease, a self-limiting infection often characterized by lymphadenopathy or by an asymptomatic course in immunocompetent patients (Eicher and Dehio, 2012; Harms and Dehio, 2012). Otherwise, *B. henselae* infections occur more frequently in immunocompromised patients (Maguiña et al., 2009) that may develop bacillary angiomatosis (BA) or peliosis (BP), vasoproliferative tumor lesions of the skin or the inner organs (Mosepele et al., 2012). Intraerythrocytic and endothelial persistence of *Bartonella* are distinguishing features in immunocompetent and immunocompromised hosts (Mosepele et al., 2012). *Bartonella* infections in the immunocompromised host may be characterized by fever, osteomyelitis, or angioproliferative lesions that may affect virtually any organ system, but have a predilection for highly vascularized tissues such a heart valves, liver, and spleen (Maguiña et al., 2009; Mosepele et al., 2012). A murine model of chronic infection in immunocompromised SCID/Beige mice showed the ability of *Bartonella* to recapitulate human pathologies; indeed, in this model, bacteria grow in extracellular aggregates, embedded within collagen matrix similar to the observations in BA, BP, and catch-scratch disease (Chiaraviglio et al., 2010). In addition, *B. henselae* can infect and damage endothelial progenitors cells (EPCs) reducing the endothelium regenerating potential (Salvatore et al., 2008, 2015; Costa et al., 2010).

*Bacteroides fragilis* is a gram-negative anaerobe bacterium belonging to the gut microflora (Coyne and Comstock, 2008). It protects mice from experimental colitis induced by *Helicobacter hepaticus* through the polysaccharide A (PSA; Mazmanian et al., 2008; Troy and Kasper, 2010). *B. fragilis*, via the PSA, can exert its effect through the immune system within both the intestinal and the systemic compartments (Mazmanian et al., 2008; Ochoa-Repàraz et al., 2010; Heijtz et al., 2011).

In a previous study, we have established that the damage induced by *B. henselae* in liver and aorta of immunocompetent mice could be prevented with *B. fragilis* wild type co-infection but not with its mutant *B. fragilis* ΔPSA (Pagliuca et al., 2012). Our earlier data showed that the prevention of damages caused by *B. henselae* was mediated by PSA, and *B. fragilis* competed with *B. henselae* during internalization of EPCs (Pagliuca et al., 2012).

In order to investigate whether *B. fragilis* can ameliorate inflammatory disease caused by *B. henselae* in an immunocompromised SCID mouse model we evaluated the effect on tissues of co-infected mice with *B. henselae* and *B. fragilis* wild type or *B. fragilis* ΔPSA by *in vivo* High-Frequency Ultrasound (HFUS) and *ex vivo* histological and Transmission Electron Microscopy (TEM) examinations. Interestingly, the scores of hepatic HFUS and histological evaluation of murine liver tissues belonging to differently infected and co-infected mice showed a substantial correlation. To the best of our knowledge this report represents the first study in a murine model of bacterial infection in which was showed the correlation between ultrasonographic and histopathological findings, but still these two techniques should be considered complementary to gain a precise diagnosis on the hepatic alterations induced by bacterial infection. In addition this is the first attempt of studying the hepatic alterations due to *B. henselae* infection and the consequent inflammation and fibrosis in immunocompromised mice with *in vivo* HFUS.

## Materials and Methods

### Bacterial Strains and Growth Conditions

*Bartonella henselae* strain ATCC49882 (LGC Promochem) was stored at -80°C in Tryptone Soya Broth USP (TSB; Oxoid) with 10% (vol/vol) glycerol (Carlo Erba) until use, and grown on Trypticase Soy Agar (TSA) with 5% sheep blood (Becton Dickinson, BD) in a humidified atmosphere at 37°C and 5% CO_2_ for 7 days. *B. fragilis* NCTC9343 wild type and its mutant *B. fragilis* ΔPSA were kindly provided by Prof. Dennis L. Kasper. The strains were stored at -80°C in Brain Heart Infusion Broth (Oxoid) with 10% (vol/vol) glycerol until use, and grown anaerobically onto BD Schaedler agar with 5% sheep blood at 37°C for 48 h.

### Murine Strain and Infection

Specific pathogens free C.B-17/IcrHsd-Prkdc^SCID^ (SCID) female mice 8 weeks of age, with severe combined immunodeficiency affecting T and B cell development, were purchased from Harlan Laboratories (Correzzana, Milano, Italy). The mice were fed with laboratory food pellets and tap water *ad libitum* and were bred and housed under specific pathogen free conditions at Ceinge-Advanced Biotechnologies, Naples, Italy. *B. fragilis* wild type or *B. fragilis* ΔPSA was given to animals (*n* = 6/group) in concentration 10^9^ CFU in 0.1 ml of Phosphate Buffer Solution (PBS) 1% per mouse by oral administration. Viable counts were performed on BD Schaedler agar with 5% sheep blood to determine the number of CFU. Mouse enteric colonization was initiated 7 days prior to *B. henselae* infection and the bacterial suspension was given as previously described (Pagliuca et al., 2012). Prior to infection, *B. henselae* was thawed at room temperature, harvested by centrifugation for 15 min at 1,500 rpm, and re-suspended in PBS 1%. Mice were infected with *B. henselae* via intra-peritoneal route at a dose of 10^9^ CFU/mouse. Control mice were injected with PBS 1% in the absence of bacteria. Viable counts were performed on TSA plates with 5% sheep blood to determine the number of CFU. Following infection, SCID mice were observed once a day for signs of illness. Every day throughout the whole experiment, animals were monitored for clinical symptoms (i.e., ruffled fur, weight loss, lethargy, or moribund). Body weight was measured by using a digital balance (Gibertini). Whenever mice showed weight loss of 30% from baseline value, euthanasia would have been applied. Since no animals showed signs of stress, no weight loss, analgesics were never given and euthanasia was used only at the end of experiment (36 days). Between 30 and 31 days post-infection, the mice were analyzed by HFUS under general anesthesia. At the times of euthanasia, animals were anesthetized (50 mg/kg ketamine and 3 mg/kg xylazine) and were humanely killed by cervical dislocation. Liver, spleen, and aorta were excised and fixed for histological and microscopic analysis. Previous observations suggested that established endpoint was able to detect tissue damage induced by *B. henselae* both in immunocompetent and immunocompromised murine host (Chiaraviglio et al., 2010; Pagliuca et al., 2012).

### Ethics Statement

All animal experiments were carried out according to institutional guidelines. All efforts were made to minimize animal suffering and to reduce the number of mice used, in accordance with the European Communities Council Directive of November 24, 1986 (86/609/EEC). The protocol of the study has been reviewed and approved by Ethical Animal Care and Use Committee of Ceinge-Advanced Biotechnologies, Naples, Italy, Prot n° 2, del 14/12/2012.

### High Frequency Ultrasound Evaluation

A total of 36 SCID mice were examined over the course of the study. All procedures were performed under general anesthesia with 4% isoflurane (induction dose) and 2% isoflurane plus oxygen (2 Lt/min; maintenance dose). Body temperature was monitored using a rectal probe and maintained in a physiological range using an infrared lamp. Hair coat on the abdominal region was removed by shaving followed by a hair remover cream, and an ultrasound-coupling gel was applied to the skin to improve ultrasound transmission and reduce contact artifacts. Mice were imaged using a transducer with a central frequency of 40 MHz (focal length 6 mm; depth of penetration ranging from 5 to 15 mm; 30–40 μm axial and 70–90 μm lateral resolution) mounted on an ultrasound system (Vevo 2100, FUJIFILM VisualSonics, Inc., Toronto, ON, Canada).

The animals were positioned in dorsal recumbency and liver images were obtained in cross-sectional two-dimensional (B-mode) imaging modality in transversal and longitudinal planes. Ultrasonographic findings were analyzed according to the following classification (Lessa et al., 2010; Resende et al., 2011): (i) Echostructure – score 0: homogeneous liver parenchyma and a regular hepatic surface; score 1: diffuse parenchymal mild heterogeneity, reduced visualization of the diaphragm and small peripheral vessels with no change on liver surface; score 2: discrete coarse and heterogeneous parenchymal echostructure, dotted or slightly irregular liver surface; score 3: extensive coarse and heterogeneous parenchymal echostructure, irregular or nodular hepatic surface with underlying regenerative nodules; (ii) Echogenicity (relative to the renal cortex) – score 0: liver less echogenic than the renal cortex; score 1: hepatic echogenicity equal to the renal cortex; score 2: liver more echogenic than the renal cortex; (iii) Presence of ascites – score 0: absent; score 1: present. Furthermore, right and caudate lobes length and caudate/right lobe ratio, the spleen area and the portal vein (PV) diameter were measured as non-invasive predictive indicator of the development of liver disease; color-Doppler and pulse-wave Doppler were used in combination to obtain the maximum PV blood velocity (Aubé et al., 1999; Tchelepi et al., 2002; Lu et al., 2003; Bonekamp et al., 2009; Resende et al., 2011).

### Histological Examination

All samples were formalin-fixed and embedded in paraffin. Serial sections, 5 μm thick were cut from each paraffin block and a section routinely stained with hematoxylin and eosin (H&E) for the morphological evaluation and grade inflammation. To assess collagen fibers, paraffin sections were stained with Masson’s Trichrome (Masson’s Trichrome AR173 Dako. Dako Autostainer Artisan Link), Sirius Red (Sirius Red/Fast Green FCF, Carlo Erba-Sigma Aldeich Direct Red 80), and Reticulin (Reticulin-Nuclear Fast Red AR179 Dako. Dako Autostainer Artisan Link). The staging of hepatic fibrosis was performed using semi-quantitative fibrosis scores (F-scores), which is similar to the score systems proposed by Batts and Ludwig (1995) and Ishak et al. (1995) for hepatic fibrosis in humans. Normal liver sections without fibrosis were classified as F0; fibrous expansion of portal areas was scored as F1; stage F2 denotes septal fibrosis with numerous marked fibrous septa. Other stages of Ishak was not observed. The portal inflammation was grading: G0 (none), G1 (mild, some or all portal areas), G2 (moderate, some or all portal areas), G3 (moderate/marked, all portal areas), G4 (marked, all portal areas) as described in literature (Ishak et al., 1995).

### Transmission Electron Microscopy (TEM)

The liver tissues were cut into 1 mm^3^ blocks and fixed in 1.5% glutaraldehyde in 0.067 M cacodylate buffer at pH 7.4 for 3 h at 4°C (Pagliuca et al., 2012). Then, they were post-fixed in 1% osmium tetroxide in 0.067 M cacodylate buffer at pH 7.4 for 1 h at 4°C and dehydrated in ascending series of ethyl alcohol and then embedded in epon. Semi-thin sections were cut with a glass knife and were stained with 1% toluidine blue for light microscopic observations. Ultra-thin sections were stained with 3% uranyl acetate in 50% ethyl alcohol and with 2.6% lead citrate and observed under a Philips EM 208 S transmission electron microscope (Philips Company) at 100 kV.

### Statistical Analysis

All tests were performed with a commercial program for statistical analysis (JMP^®^ 8.0, SAS Institute Inc., Cary, NC, USA). All measurements described in the ultrasonographic evaluation were compared between the infected groups and the PBS control group with the Dunnett’s test; a group received a score of 1 if a significant difference was detected. The partial scores of the echostructure, echogenicity, and total scores were compared with the PBS control group using the Dunnett’s test. Furthermore, both scores and measurements were compared between all groups using “each couple” Student’s *t-*test. The ultrasound scores were correlated with the histological scores with the Spearman’s rank-order correlation coefficient (r_s_). The limit of significance was set at *p* < 0.05 (Dytham, 2011; Petrie and Watson, 2013).

## Results

### Immunocompromised Murine Infection Model

**FIGURE 1:**
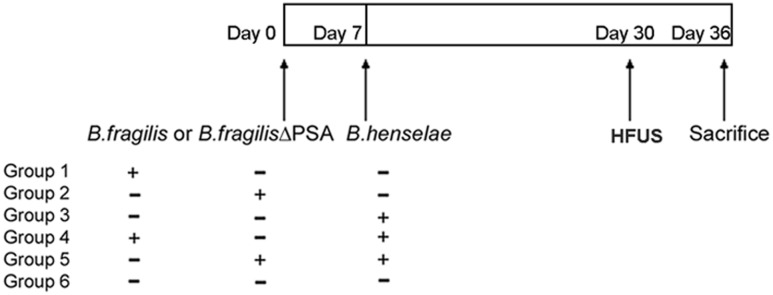
**Murine infection with *Bartonella henselae* and *Bacteroides fragilis*.** Scheme of immunocompromised murine infection: three groups of SCID mice (*n* = 6/group) were infected with *B. henselae*, or *B. fragilis*, or *B. fragilis* ΔPSA (polysaccharide A), two groups of animals (*n* = 6/group) were co-infected with *B. henselae* and *B. fragilis* or *B. fragilis* ΔPSA, and one group were injected with Phosphate Buffer Solution (PBS) 1% in the absence of bacteria (PBS control group).

In order to determine whether PSA was essential for protection against pathogenic effects induced by *B. henselae*, SCID mice were inoculated with either *B. fragilis* wild type or its mutant *B. fragilis* ΔPSA. Three groups of six immunocompromised mice were infected with 10^9^ CFU of either *B. fragilis* wild type or *B. fragilis* ΔPSA or *B. henselae*, respectively (Groups 1–3), whereas two groups of animals were co-infected with *B. henselae* (10^9^ CFU) and alternatively with *B. fragilis* wild type (Group 4) or *B. fragilis* ΔPSA (10^9^ CFU; Group 5). The control was represented by Group 6 (**Figure 1**). The health of animals was evaluated once a day for the full duration of the experiment, no animals showed sign of suffering or illness (data not show) and no animals died after any infection. Survival was recorded for the whole duration of experiment. After 30 days, the animals were analyzed by HFUS and, after 36 days, mice were euthanized in order to assess both the bacterial infections and the role of *B. fragilis* PSA by histopathology and TEM evaluation on infected tissues.

### High Frequency Ultrasound Evaluation

The morphological, functional and echostructural analysis of the liver performed with HFUS demonstrated clearly that *B. henselae* infected mice as well as *B. henselae*/*B. fragilis* ΔPSA co-infected mice showed most severe signs of liver disease in comparison with those co-infected with *B. henselae*/*B. fragilis* wild type and no infected (**Table 1**; **Supplementary Figure S1**). Nevertheless, none of the measurement obtained with HFUS resulted to be different between the infected and control SCID mice, nor among the different types of infection (Supplementary Table S1).

**Table 1:** High frequency ultrasound scores of echostructure, echogenicity, and ascites.

Group	Echostructure	Echogenicity	Ascites	Total
*B. henselae*	2^A^	0.5	0.5	3^Aa^
*Bartonella henselae/Bacteroides fragilis*	1.5^a^	0	0	1.5^b^
*B. fragilis*	1.5^a^	0	0	1.5^b^
*B. fragilis*ΔPSA (polysaccharide A)	1.5^a^	0	0	1.5^b^
*B. henselae/B. fragilis*ΔPSA	2^A^	1^a^	0	3^Aa^
Control	0^Bb^	0^b^	0	0^Bc^


*Median value of scores assigned to echostructure, echogenicity, ascites, and the total score. Statistical differences: A > B, *p* < 0.01; a > b, b > c, *p* = 0.01.*

**FIGURE 2:**
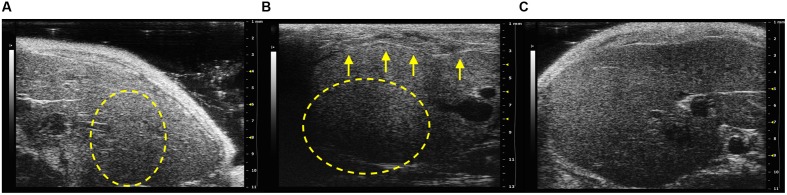
**Analysis of hepatic echostructure of infected SCID mice.** **(A)** Example of score 1: diffusely increased parenchymal echogenicity, reduced visualization of the diaphragm and small peripheral vessels (circular dotted line) with no change on liver surface in SCID co-infected mice with *B. henselae*/*B. fragilis* wild type. **(B)** Example of score 2: discrete coarse and heterogeneous parenchymal echogenicity (circular dotted line), dotted or slightly irregular liver surface in SCID co-infected mice with *B. henselae*/*B. fragilis*ΔPSA (arrows). **(C)** Example of score 0: homogeneous liver parenchyma with medium level echogenicity and a regular hepatic surface in SCID PBS control group.

All infected mice presented an altered echostructure, which resulted significantly different compared with the PBS control group (*p* < 0.05; **Figure 2C**). An example of the different scores of hepatic echostructure are shown in **Figure 2**. In particular, SCID mice infected with *B. henselae* and co-infected with *B. henselae/B. fragilis* ΔPSA presented a discrete coarse and heterogeneous parenchymal echogenicity (Example of score 2; **Figure 2B**) compared with those co-infected with *B. henselae/B. fragilis* wild type that presented a diffusely increased parenchymal echogenicity on liver surface (Example of score 1; **Figure 2A**).

**FIGURE 3:**
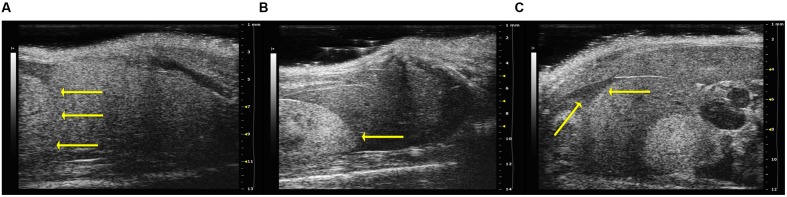
**Analysis of hepatic echogenicity of infected SCID mice.** In the figure are shown examples of echogenicity, relative to the renal cortex, (arrow)-on the left of the pictures. **(A)** Example of score 1: hepatic echogenicity equal to the renal cortex observed in SCID co-infected mice with *B. henselae*/*B. fragilis*ΔPSA. **(B)** Example of score 0: liver less echogenic than the renal cortex (arrow) in SCID co-infected mice with *B. henselae*/*B. fragilis* wild type. **(C)** Presence of ascites (on the arrow), caval and portal dilatation (on the right of the picture) in SCID mice infected with *B. henselae.*

Alteration of echogenicity was noted in the 50% of the mice infected with *B. henselae* and in all *B. henselae/B. fragilis*ΔPSA co-infected mice (Example of score 1; **Figure 3A**), characterized by hepatic echogenicity equal to the renal cortex. In all other mice the liver was less echogenic than the renal cortex (Example of score 0; **Figure 3B**). The echogenicity score of co-infected mice with *B. henselae/B. fragilis* ΔPSA differed significantly compared with the PBS control group (*p* < 0.05). In addition ascites, with an accumulation of fluid in the abdominal cavity, was detected in 50% of the mice infected with *B. henselae* (**Figure 3C**) but no statistical difference was detected compared with the PBS control group. The median scores of echostructure, echogenicity, ascites, and the total scores (i.e., the sum of the scores above mentioned) for each group are reported in **Table 1**. The HFUS evaluation revealed alterations of the liver parenchyma but did not show any pathological features in the other observed structures, including small and large bowel (data not shown).

The median total HFUS scores were significantly higher in mice infected with *B. henselae* and co-infected mice with *B. henselae/B. fragilis* ΔPSA compared to all other groups (*p* < 0.01), whereas all other infected mice groups had a higher score compared to PBS control mice (*p* = 0.01; **Table 1**; **Supplementary Figure S1**).

### Histopathological Analysis and Correlation with HFUS Evaluation

**FIGURE 4:**
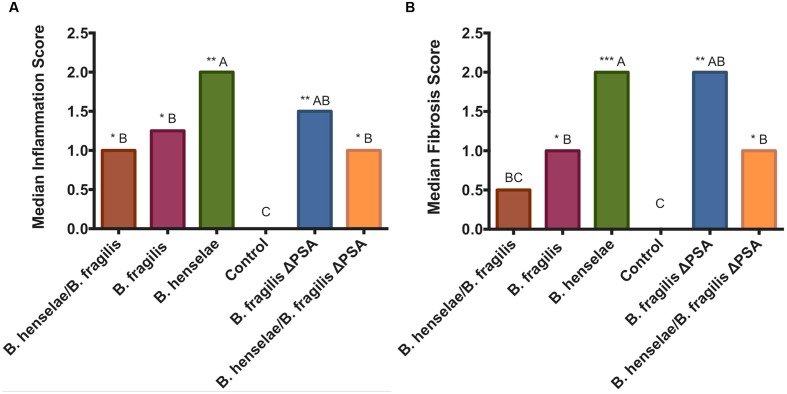
**Graphic representation of the median scores of the histopathological grading.** **(A)** Bar graph represents the median inflammation score in the different groups. **(B)** Bar graph represents the median fibrosis score in the different groups. Statistical differences: A > B, B > C. ^∗∗∗^*p* < 0.001, ^∗∗^*p* < 0.01, ^∗^*p* < 0.05.

**FIGURE 5:**
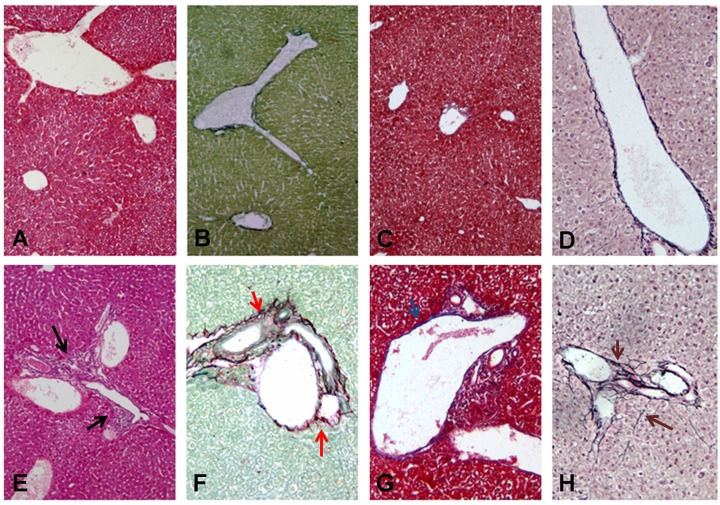
**Histological analysis of liver sections in infected SCID mice with *B. henselae* compared with PBS control group.** **(A–D)** Representative liver section showing G0 **(A)** H&E ×10 magnification, black arrow and F0 **(B)** Sirius Red/Fast Green ×10 magnification, red arrow; **(C)** Masson’s Trichrome ×10 magnification, blue arrow; **(D)** Reticulin-Nuclear Fast Red ×20 magnification, brown arrow in the PBS control group. **(E–H)** Representative liver section showing G2 **(E)** H&E ×10 magnification, black arrow and F2 **(F)** Sirius Red/Fast Green ×10 magnification, red arrow; **(G)** Masson’s Trichrome ×10 magnification, blue arrow; **(H)** Reticulin-Nuclear Fast Red ×20 magnification, brown arrow in the *B. henselae* infected group.

**FIGURE 6:**
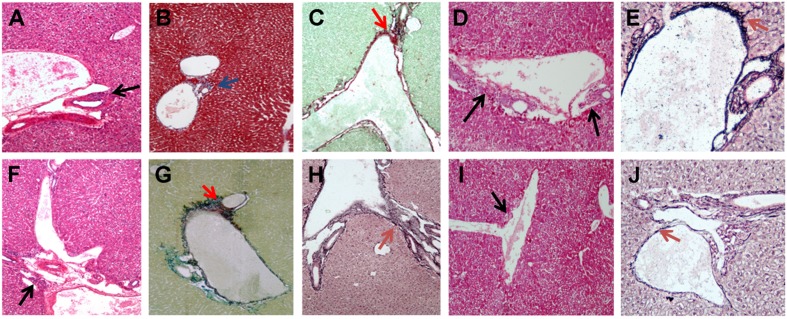
**Histological analysis of liver sections in infected SCID mice with *B. fragilis. B. fragilis* △PSA, and in co-infected SCID mice.** **(A–C)** Representative liver section showing G1 **(A)** H&E ×10 magnification, black arrow, and F1 **(B)** Masson’s Trichrome ×10 magnification, blue arrow; **(C)** Sirius Red/Fast Green ×10 magnification, red arrow in the SCID co-infected mice with *B. henselae/B. fragilis* wild type; **(D,E)** Representative liver section showing G1–G2 **(D)** H&E ×10 magnification, black arow and F1 **(E)** Reticulin-Nuclear Fast Red ×20 magnification, brown arrow in the *B. fragilis* △PSA infected group. **(F–H)** Representative liver section showing G1 **(F)** H&E ×10 magnification, black arrow and F1 **(G)** Sirius Red/Fast Green ×10 magnification, red arrow; **(H)** Reticulin-Nuclear Fast Red ×20 magnification, brown arrow in the *B. fragilis* wild type infected group; **(I,J)** Representative liver section showing G1 **(I)** H&E ×10 magnification, black arrow and F1–F2 **(J)** Reticulin-Nuclear Fast Red ×20 magnification, brown arrow in the *B. henselae/B. fragilis* △PSA infected group.

After 36 days, animals were euthanized in order to assess both the effect of bacterial infection and *B. fragilis* protection by histopathology and TEM of infected tissues (see paragraphs below). The median scores for hepatic phlogosis and fibrosis obtained by histopathological evaluation are reported in **Figure 4**. All infected SCID mice showed a phlogosis and fibrosis score (**Figures 5** and **6**) significantly higher compared with the PBS control group (**Figures 5A–D**). In particular, it was highlighted by H&E staining an inflammation of G2 score (moderate, some or all portal areas) in SCID mice infected with *B. henselae* (**Figure 5E**), which resulted significantly higher compared to all other groups (*p* ≤ 0.01), except for *B. fragilis*ΔPSA (**Figure 4**). A moderate inflammation, of G1–G2 score, was found in animals infected with *B. fragilis*ΔPSA (**Figure 6D**). Instead, a portal inflammation of G1 score (mild, some or all portal areas) was observed both in mice infected with *B. fragilis* wild type (**Figure 6F**) and in both co-infected groups (**Figures 6A,I**). In addition, mice infected with *B. henselae* and co-infected with *B. henselae/B. fragilis*ΔPSA also had the higher fibrosis score equal to F2 (**Figures 4**, **5H**, and **6J**), whereas all other infected mice exhibited a fibrosis score ≤ to F1 (fibrous expansion of portal areas; **Figures 6B,C,E,G,H**) compared with the PBS control group (*p* < 0.05; **Figures 4** and **5B–D**). Histopathological data confirmed that SCID mice infected with *B. henselae* showed the highest total score (**Figure 4**).

The statistical analysis performed on the whole HFUS and histopathological dataset showed a significant positive correlation between the inflammation score and the echostructure (*rs* = 0.63; *P* = 0.020), ascites (*rs* = 0.55; *P* = 0.048), and the total scores (*rs* = 0.65; *P* = 0.017), furthermore the fibrosis score had a significant correlation with echostructure (*rs* = 0.61; *P* = 0.026), ascites (*rs* = 0.49; *P* = 0.048), and total scores (*rs* = 0.62; *P* = 0.025). Considering the mean scores for each group, the same correlations were confirmed, and a further correlation between fibrosis and echogenicity scores was detected (*rs* = 0.35; *P* = 0.038).

The histopathological analysis carried out on other organs (spleen and aorta) showed no pathological alteration (data not shown).

### Ultrastructural Analysis

**FIGURE 7:**
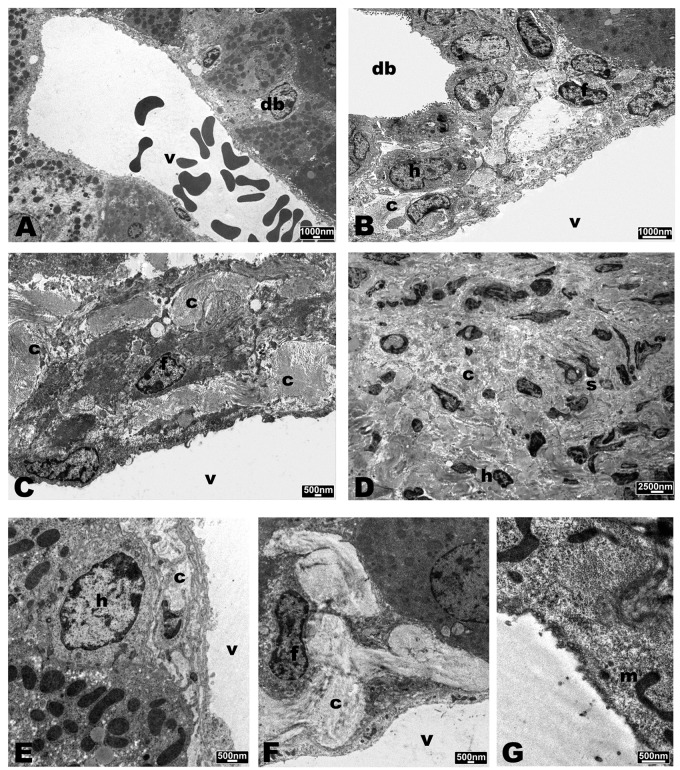
**Transmission Electron Microscopy (TEM) micrographs of liver of SCID mice.** **(A)** PBS control group. **(B)** Mice infected with *B. fragilis* wild type. **(C)** Mice infected with *B. fragilis* ΔPSA. **(D)** mice infected with *B. henselae*. **(E)** co-infected mice with *B. henselae/B. fragilis* wild type. **(F,G)** Co-infected mice with *B. henselae/B. fragilis* ΔPSA. **(A)** Portal triad with a small bile duct (db) and the portal vein (v). Hepatocytes with rounded nucleus and massive presence of mitochondria and peroxisomes; **(B)** portal triad with bile duct (db) and the portal vein (v). Slight amount of collagen (c) and histiocytic cells (h), fibroblasts (f); **(C)** portal vein (v) with massive presence of collagen (c) below the endothelium; **(D)** macro-area rich in collagen (c) and stellate cells (s); **(E)** portal vein (v) with slight amount of collagen under the endothelium and histiocytic cell (h); **(F)** portal vein (v) with considerable amount of collagen below the endothelium and fibroblast (f); **(G)** altered endothelial cell with ribbon-mitochondria (m).

**FIGURE 8:**
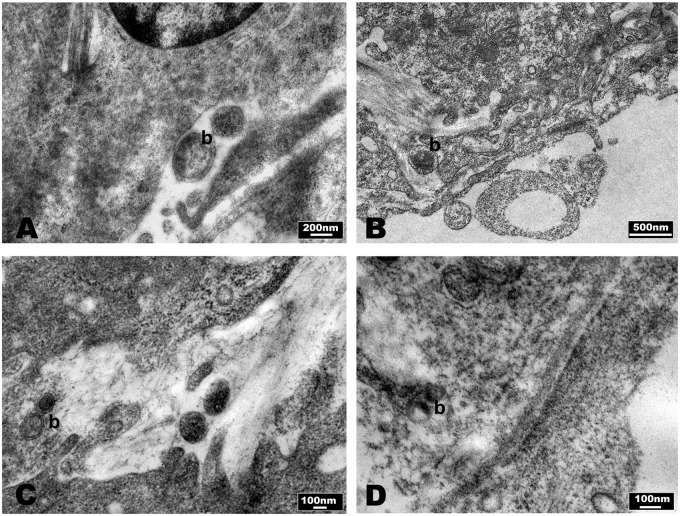
**Evaluation of bacteria in SCID mice liver by TEM micrographs.** **(A,B)** Mice infected with *B. henselae*, **(C)** co-infected mice with *B. henselae/B. fragilis* wild type, **(D)** co-infected mice with *B. henselae/B. fragilis* ΔPSA. **(A)** *B. henselae* (b) within area rich in collagen; **(B)** *B. henselae* (b) inside space of Disse; **(C)** *B. henselae* (b) inside space of Disse; **(D)** *B. henselae* (b) between the fibers of collagen in space of Disse.

Ultrastructural analyses performed by TEM revealed no significant alterations in liver samples of mice infected with *B. fragilis* wild type (**Figure 7B**) compared with the PBS control group (**Figure 7A**) except for the presence of histiocytic cells in the sub-endothelial region of the main vessels. In samples infected with *B. fragilis* ΔPSA the presence of collagen around the main vessels was observed (**Figure 7C**). The liver samples of mice infected with *B. henselae* (**Figure 7D**) showed macro-areas rich in collagen, stellate cells, and histiocytic cells. Rare *B. henselae* were found in collagen (**Figures 8A,B**). The liver samples co-infected with *B. henselae*/*B. fragilis* wild type showed the presence of histiocytic cells in the sub-endothelial region of the main vessels (**Figure 7E**). Here, limited areas with collagen containing these bacteria were also found (**Figure 8C**). In liver samples co-infected with *B. henselae*/*B. fragilis* ΔPSA, the presence of areas rich in collagen in the sub-endothelial region of the main vessels and adjacent to hepatocytes were observed (**Figure 7F**). These areas were smaller compared with those of liver samples infected only with *B. henselae* (**Figure 7D**). Bacterial internalization was observed in these samples (**Figure 8D**). The endothelial cells of the main vessels presented morphological alterations with ribbon mitochondria (**Figure 7G**) and high amount of smooth reticulum profiles. In all samples analyzed for each infection type, no alterations either hepatocytes or bile ducts, or sinusoids were found.

## Discussion

The results of this study have established, for the first time, that HFUS evaluation showed the ability to identify echostructural alterations in all infected SCID mice compared with PBS control group. Moreover our *in vivo* studies indicated that half of mice infected with *B. henselae* and all co-infected mice with *B. henselae/B. fragilis* ΔPSA showed an altered echogenicity with the hepatic echogenicity equal to the renal cortex. It is important to underline that, by this approach, kidney diseases should be excluded (Resende et al., 2011). Moreover, ultrasonography allowed to confirm the presence of ascites, even when mild, another sign of portal hypertension and diffuse liver disease (Heller and Tublin, 2014). HFUS evaluations demonstrated that *B. henselae* infected mice and *B. henselae/B. fragilis* ΔPSA co-infected mice showed the most severe morpho-structural signs of liver disease. These results suggest that damages induced by *B. henselae* can be reduced, on the HFUS point of view, by the colonization with *B. fragilis*, but not by *B. fragilis* ΔPSA, supporting the ultrastructural data previous reported (Pagliuca et al., 2012).

Interestingly, to date there is no standardized scoring/grading system for liver fibrosis and/or inflammation observed in mice by HFUS. Some authors evaluated the sensitivity, the specificity, and the predictive values of liver echogenicity by HFUS compared with the renal cortex, and the echostructure and the PV diameter in rat models of chronic hepatic disease (Lessa et al., 2010). In our study, we included also other HFUS measurements evaluating the ultrasonographic diagnosis of hepatic fibrosis and cirrhosis in human as previously reported by Aubé et al. (1999). Indeed, in humans, the ratio of the caudate and the right hepatic lobe was used to diagnose cirrhosis more effectively than a measurement of a single lobe size (Tchelepi et al., 2002). Portal vein diameter and velocity were reported as a marker of human portal hypertension but they could not be used to differentiate the degree of hepatitis and cirrhosis (Gerstenmaier and Gibson, 2014).

In our study, the quantitative ultrasonographic measurements did not differ between the infected and the PBS control group. On the other hand, the qualitative scoring system applied allowed to find significant differences between the different types of infections. It should be considered that both the quantitative and qualitative applied in our study have been extrapolated from numerous pre-clinical and clinical reports (Aubé et al., 1999; Mathiesen et al., 2002; Lu et al., 2003; Bonekamp et al., 2009; Lessa et al., 2010; Resende et al., 2011; Chen et al., 2013). Since this is the first application in a murine model of bacterial infection, the lack of significance might be more related to the need of a diversified diagnostic approach between different species than to the absence of pathologic ultrasonographic features.

Various reports have evaluated the sensitivity and specificity of non-invasive diagnostic aids (Lu et al., 2003; Bonekamp et al., 2009) but the diagnosis of liver fibrosis still depends on pathological examination of liver puncture tissue. Different studies in humans and experimental rodents model tried to correlate the degree of steatosis, fibrosis, altered liver enzymes levels, and ultrasonographics findings, achieving quite similar results (Mathiesen et al., 2002; Guimond et al., 2007; Yan et al., 2007). Recently, transient and real-time elastography are being used to assess more objectively and accurately tissue elasticity but they showed to be reliable only for extensive fibrosis or cirrhosis (Bonekamp et al., 2009; Gerstenmaier and Gibson, 2014; Heller and Tublin, 2014). Most pre-clinical studies focused on steato-fibrosis have used cirrhosis models induced with different chemicals and/or bile duct ligation, or with genetically engineered (Lee et al., 2005; Guimond et al., 2007; Yan et al., 2007; Lessa et al., 2010; Hayashi and Sakai, 2011; Chen et al., 2013).

Our results showed a substantial relationship between the ultrasonographic and histological findings, confirmed by the statistical correlation amongst the scores assigned by the two techniques. Many scientific reports tried to explore whether a correlation exists between ultrasonographic and histopathological indexes, but the results vary and sometimes contradict (Aubé et al., 1999; Mathiesen et al., 2002; Bonekamp et al., 2009; Lessa et al., 2010), especially considering that different histological grading/staging approaches for hepatitis exist (Ishak et al., 1995; Theise, 2007; Bonekamp et al., 2009). Nevertheless, the two techniques should still be considered as complementary: the ultrasound examination cannot detect signs of ultrastructural liver disease caused by *B. henselae*, whereas it is ideal to identify the presence of ascites, the dilatation of the hepatic veins and portal vessels, and to evaluate the liver surface or the presence of nodularity caused by infective agents.

To the best of our knowledge, this is the first attempt of studying the hepatic alterations caused by bacterial infection and the consequent inflammation and fibrosis with HFUS. In fact, both healing and fibrotic processes, in response to inflammation, lead to deposition of extracellular matrix and hence to fibrosis (Hayashi and Sakai, 2011). Imaging methodologies, including ultrasonography, play an important role in the detection, characterization, and management of infectious liver diseases in human clinical practice. Bacterial abscesses and parasitic, fungal and viral diseases have more or less specific imaging findings that together with appropriate clinical information may provide the most likely diagnosis.

In human beings, *B. henselae* hepatitis shows non-specific ultrasonographic signs, i.e., round hypoechoic lesions (Mortelé et al., 2004). In our model of infection in SCID mice infected with *B. henselae*, we were able to detect a series of ultrasonographic features linked to the inflammatory/fibrotic response. In addition, ultrasonographic features of co-infected mice both with *B. henseale*/*B. fragilis* and *B. henselae/B. fragilis* ΔPSA, in accordance with histopathological analysis, have confirmed that inflammatory liver damage caused by *B. henselae* can be prevented by *B. fragilis* colonization (Pagliuca et al., 2012). In *B. henselae* infected mice and co-infected mice with *B. fragilis* ΔPSA mutant we also documented a increased presence of septal fibrosis with numerous marked fibrous septa completely recapitulate by the co-infection with *B. fragilis* wild type. This finding corroborates the hypothesis that PSA is involved in the process of immunoregulation and exert a protective effect in murine host (Mazmanian et al., 2005, 2008; Pagliuca et al., 2012).

It is noteworthy that the absence of dead mice and ultrastructural data showed that SCID mice tolerated *B. henselae* infection at high concentrations in agreement with Chiaraviglio et al. (2010). This tolerance was in contrast with other Gram-negative bacteremia where without a rapid antibiotic therapy the death followed quickly the infection (Barquero-Calvo et al., 2007; Andonegui et al., 2009; Roger et al., 2009). SCID mice, lacking of mature lymphocytes, do not respond to *B. henselae* infection with the formation of granulomas as observed in immunocompetent mice (Pagliuca et al., 2012) but with a considerable deposition of collagen in the region surrounding the centrilobular vein and mostly in the portal region of the liver, a characteristic found also in human diseases such as BP and BA (Cockerell et al., 1991; Borczuk et al., 1998). The deposition of collagen would seem to be due not only to portal fibroblasts but also to the massive presence of active hepatic stellate cells that lost their characteristic cytoplasmic drops containing retinoids (Friedman et al., 1985). Indeed, these cells presented on their membrane Toll like receptor-2 (TLR-2) and TLR-4 receptors, which are able to recognize and respond to various pathogen associated molecular patterns (Brun et al., 2005), including PSA (Surana and Kasper, 2012).

The mice infected with *B. fragilis* wild type had only a mild inflammation while those infected with *B. fragilis* ΔPSA had a minimal presence of collagen around the main vessels that might be considered physiological. In liver samples of animals co-infected with *B. henselae*/*B. fragilis* wild type a reduction of collagen and a higher histiocytic response was observed with respect to the mice co-infected with *B. henselae*/*B. fragilis* ΔPSA. The increase of histiocytic response was also observed in the liver of immunocompetent mice infected with *B. henselae* or co-infected with *B. fragilis* wild type (Pagliuca et al., 2012). This further observation supports the hypothesis that, in immunocompromised condition, the PSA, recognized by TLR-2, stimulates the proliferation and activation of innate immunity cells. Instead in case of co-infections, determines restraints fibrosis, caused by *B. henselae*, through production of the anti-inflammatory cytokine IL-10 (Cohen-Poradosu et al., 2011). The alterations found in endothelial cells of samples co-infected with *B. henselae*/*B. fragilis* ΔPSA could be due, as it is well known, to the ability of *B. henselae* to internalize endothelial cells (Dehio et al., 1997; Salvatore et al., 2008).

## Conclusion

These data corroborate the hypothesis that liver injury induced by *B. henselae* infection can be reduced by *B. fragilis* PSA in an immunocompromised murine model. Interestingly, in this study it was established, for the first time, that the HFUS, in combination with histopathological evaluation, could be considered good imaging approach to evaluate hepatic alterations in experimental murine model.

## Author Contributions

All authors listed have made substantial, direct and intellectual contribution to the work, and approved it for publication PS and BA conceived the study. PS, BC, CP, RC, and AG wrote the manuscript. AG, LA, and SA performed ultrasound experiments and statistical analysis. CP, AGC, RC, GP, and ES performed murine model of infection and microbiological experiments. GM performed histological examination. BA and RaC performed TEM experiments. CaP and AB contributed to experimental data and contributed to the manuscript.

## Conflict of Interest Statement

Neither the submitted paper nor any similar paper has been or will be submitted to or published in any primary scientific journal. All authors are aware of and agree to the content of the paper and their being listed as authors on the paper.

The authors declare that the research was conducted in the absence of any commercial or financial relationships that could be construed as a potential conflict of interest.

## Supplementary Material

**FIGURE S1 | Graphic representation of the median scores of the total HFUS grading.** Bar graph represents the median scores of the total HFUS evaluation. Statistical differences: A > B, B > C. ^∗∗∗^*p* < 0.001, ^∗∗^*p* < 0.01.
