# Supplementary figures and images for: Novel Approach for Evaluation of Bacteroides fragilis Protective Role against Bartonella henselae Liver Damage in Immunocompromised Murine Model
Source: Front Microbiol. 2016 Nov 7;7:1750. doi: 10.3389/fmicb.2016.01750 (PMC5097911; doi:10.3389/fmicb.2016.01750)

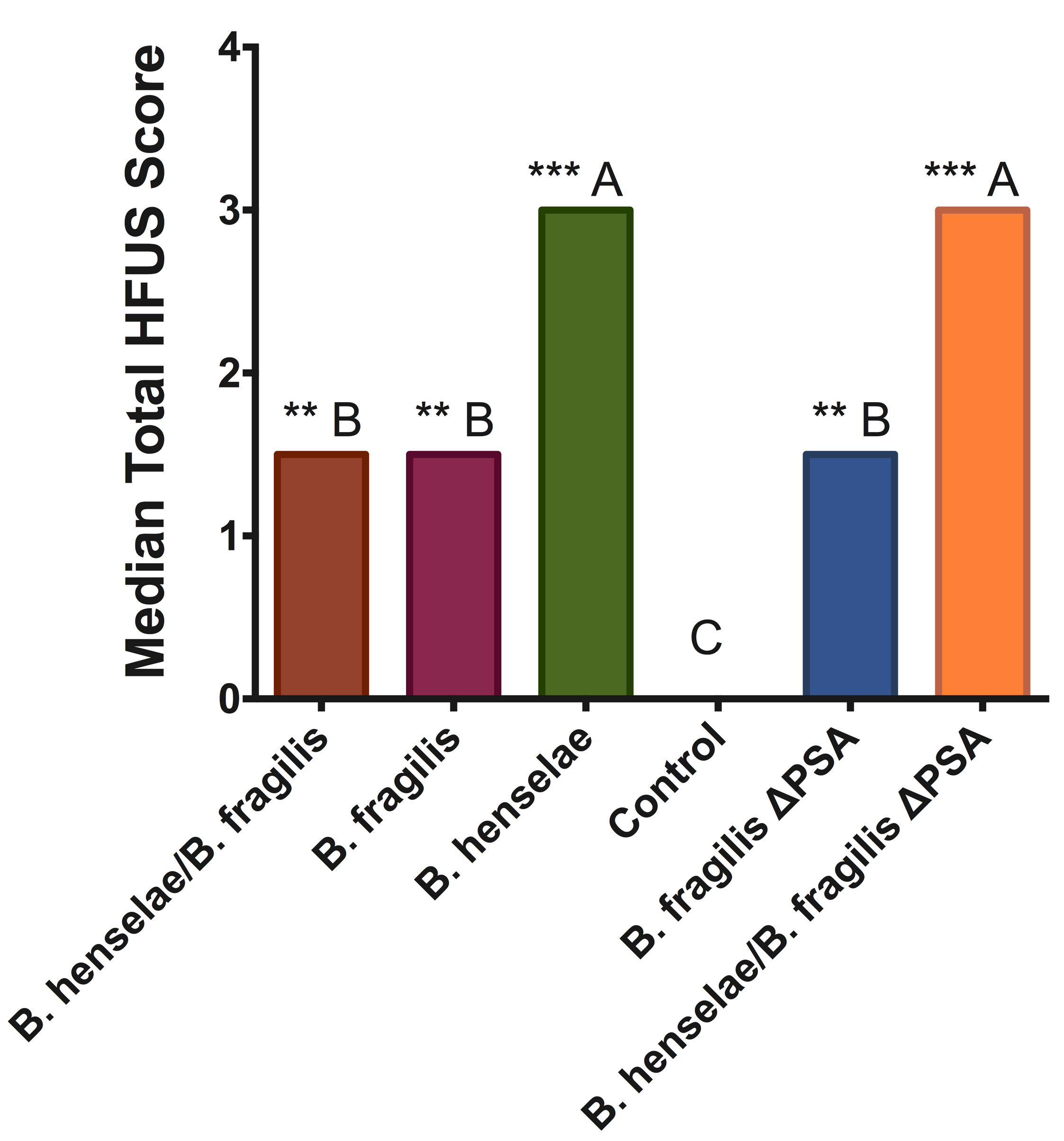

Supplement: Supplementary file 1 [file Image_1.TIFF]
