# Supplementary material for: Novel Approach for Evaluation of Bacteroides fragilis Protective Role against Bartonella henselae Liver Damage in Immunocompromised Murine Model
Source: Front Microbiol. 2016 Nov 7;7:1750. doi: 10.3389/fmicb.2016.01750 (PMC5097911; doi:10.3389/fmicb.2016.01750)
Supplement: Supplementary file 2 [file Table_1.DOCX]

| **Group** | **Rigth Lobe** | **Caudate Lobe** | **Caudate/Right** | **PV diameter** | **Max PV velocity** | **Spleen area** |
| --- | --- | --- | --- | --- | --- | --- |
| ***B. henselae*** | 7.08±1.05 | 4.12±0.04 | 0.57±0.13 | 1.12±0.16 | 38.98±13.53 | 10.51±4.00 |
| ***B. henselae/B. fragilis*** | 7.49±2.06 | 7.57±3.35 | 0.72±0.27 | 1.14±0.18 | 30.16±25.89 | 8.93±3.70 |
| ***B. fragilis*** | 9.37±1.45 | 5.59±0.02 | 0.54±0.01 | 1.26±0.15 | 39.01±7.34 | 12.09±5.45 |
| ***B. fragilis ∆*PSA** | 9.99±1.98 | 5.58±0.19 | 0.54±0.06 | 1.13±0.12 | 17.50±3.68 | 31.71±14.89 |
| ***B. henselae/B. fragilis* ΔPSA** | 11.81±1.50 | 7.57±3.08 | 0.64±0.23 | 1.06±0.46 | 20.34±6.39 | 31.74±11.86 |
| **Control** | 9.60±0.69 | 6.13±1.43 | 0.91±0.19 | 0.97±0.07 | 42.17±16.05 | 12.41±3.70 |

**Supplementary Table 1. HFUS morphormetric and functional parameters in the liver disease.**

Mean ± SD of the HFUS measurements: right and caudate lobes length and the caudate/right lobe ratio, the portal vein (PV) diameter, maximum portal vein blood velocity (Max PV velocity) and the spleen area were measured.
